# The molecular determinants regulating redox signaling in diabetic endothelial cells

**DOI:** 10.3389/fphar.2025.1563047

**Published:** 2025-04-01

**Authors:** Swayam Prakash Srivastava, Olivia Kopasz-Gemmen, Aaron Thurman, Barani Kumar Rajendran, M. Masilamani Selvam, Sandeep Kumar, Rohit Srivastava, M. Xavier Suresh, Reena Kumari, Julie E. Goodwin, Ken Inoki

**Affiliations:** ^1^ Life Sciences Institute, University of Michigan, Ann Arbor, MI, United States; ^2^ Department of Pediatrics, Yale University School of Medicine, New Haven, CT, United States; ^3^ Vascular Biology and Therapeutic Program, Yale University School of Medicine, New Haven, CT, United States; ^4^ Department of Pathology, Yale School of Medicine, Yale University, New Haven, CT, United States; ^5^ Department of Pharmaceutical Technology, Paavai Engineering College, Namakkal, Tamil Nadu, India; ^6^ Department of Cellular Biology and Anatomy, Augusta University, Augusta, GA, United States; ^7^ Laboratory of Medical Transcriptomics, Department of Endocrinology, Nephrology Services, Hadassah Hebrew-University Medical Center, Jerusalem, Israel; ^8^ School of Advanced Sciences and Languages, VIT Bhopal University, Sehore, Madhya Pradesh, India; ^9^ Department of Physiology, Augusta University, Augusta, GA, United States; ^10^ Department of Molecular and Integrative Physiology, University of Michigan, Ann Arbor, MI, United States; ^11^ Department of Internal Medicine, Division of Nephrology, University of Michigan Medical School, Ann Arbor, MI, United States

**Keywords:** diabetes, endothelial cells, EndMT, AMPK, mTORC1, endothelial dysfunctions

## Abstract

Oxidation and reduction are vital for keeping life through several prime mechanisms, including respiration, metabolism, and other energy supplies. Mitochondria are considered the cell’s powerhouse and use nutrients to produce redox potential and generate ATP and H_2_O through the process of oxidative phosphorylation by operating electron transfer and proton pumping. Simultaneously, mitochondria also produce oxygen free radicals, called superoxide (O_2_^−^), non-enzymatically, which interacts with other moieties and generate reactive oxygen species (ROS), such as hydrogen peroxide (H_2_O_2_), peroxynitrite (ONOO−), and hydroxyl radical (OH^−^). These reactive oxygen species modify nucleic acids, proteins, and carbohydrates and ultimately cause damage to organs. The nutrient-sensing kinases, such as AMPK and mTOR, function as a key regulator of cellular ROS levels, as loss of AMPK or aberrant activation of mTOR signaling causes ROS production and compromises the cell’s oxidant status, resulting in various cellular injuries. The increased ROS not only directly damages DNA, proteins, and lipids but also alters cellular signaling pathways, such as the activation of MAPK or PI3K, the accumulation of HIF-1α in the nucleus, and NFkB-mediated transcription of pro-inflammatory cytokines. These factors cause mesenchymal activation in renal endothelial cells. Here, we discuss the biology of redox signaling that underlies the pathophysiology of diabetic renal endothelial cells.

## 1 Introduction

**FIGURE 1:**
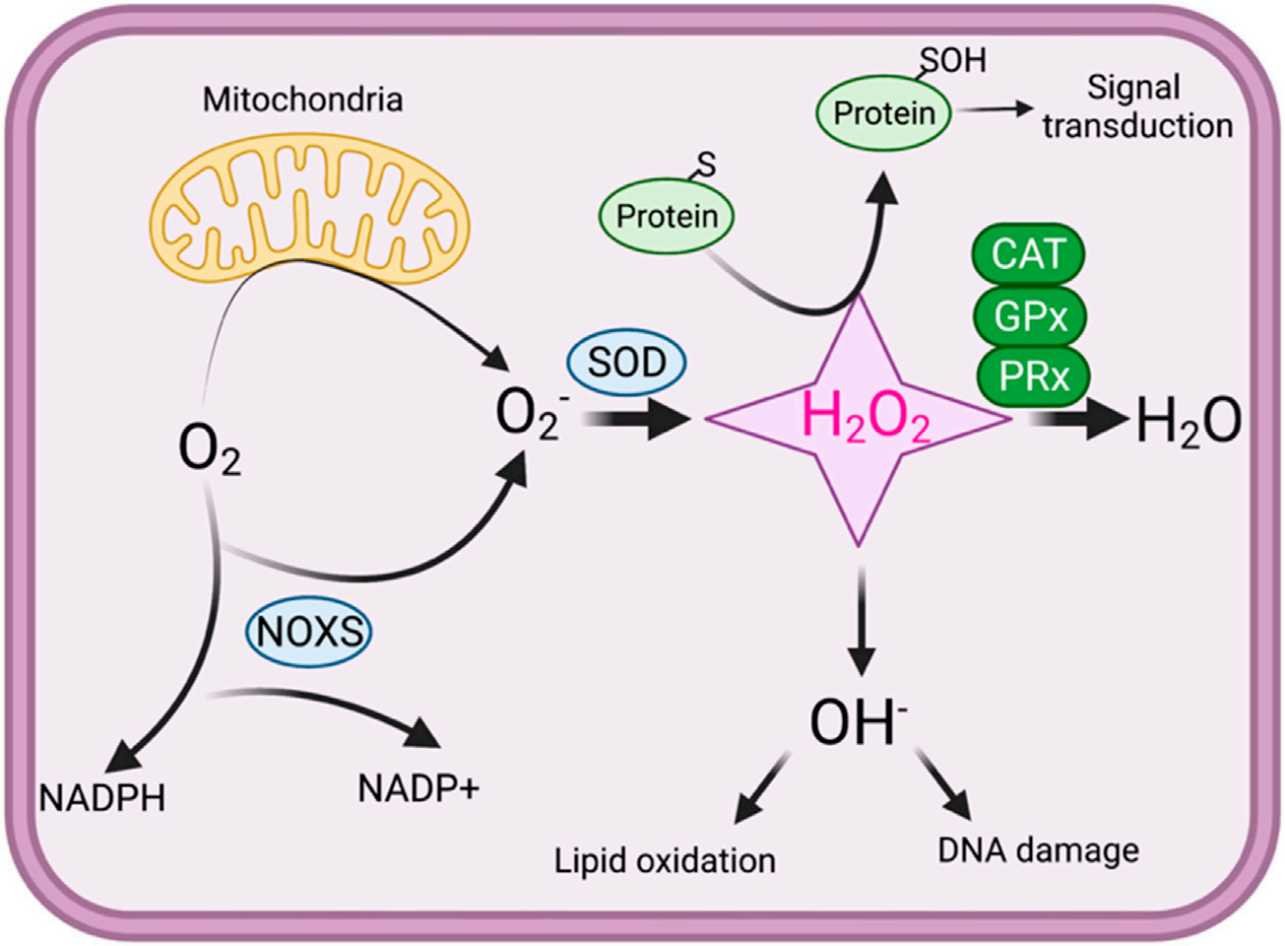
An overview of redox signaling. In oxidative phosphorylation, mitochondria use oxygen as an electron acceptor, producing superoxide (O_2_·−). Superoxide is then converted into hydrogen peroxide (H_2_O_2_), which can either be further reduced to water through the action of catalase (CAT), glutathione peroxidase (GPx), and peroxiredoxins (PRx) or transformed into harmful reactive oxygen species (ROS). Hydroxyl radicals (·OH^−^) can cause defects in the lipid oxidation and lead to DNA damage. Under physiological conditions, protein thiol exist in the thiolate anion (S−) state, making them more susceptible to oxidation by H_2_O_2_. H_2_O_2_ oxidizes the thiolate anion, forming sulfenic acid, which can react with other thiols to generate disulfide bonds. In high concentrations, H_2_O_2_ can further oxidize sulfenic acid into sulfinic acid, potentially resulting in oxidative stress.

Oxidative stress is produced by a loss of control over reactive oxygen species (ROS) production and a biological system’s capability to equilibrate the reactive species and related cellular injuries. Disruptions in the regular redox state of cells result in adverse effects via enhanced production of reactive free radicals, which harms cell organelles (Liguori et al., 2018; Pizzino et al., 2017; Nita and Grzybowski, 2016). ROS are derivatives of aerobic metabolism (Lennicke and Cocheme, 2021a; An et al., 2024). Superoxide anion (O_2_^−^), hydrogen peroxide (H_2_O_2_), and hydroxyl radicals (OH^·^) are components of ROS; all these have natural biochemical reactivity to other biological objects (Lennicke and Cocheme, 2021a; An et al., 2024). ROS are regularly linked with oxidative stress machinery, which proposes that ROS prompts pathology by burning lipids, DNA, and proteins (Schieber and Chandel, 2014) (Figure 1).

Mitochondria are the primary source of ROS production in both physiological and pathological conditions (Pizzino et al., 2017; Al-Gubory et al., 2012; Russell et al., 2020). Although these organelles are involved in intrinsic ROS scavenging capability (Hansen et al., 2006; Glasauer and Chandel, 2014). ROS also serve as signaling molecules that help in the regulation of biological processes (Schieber and Chandel, 2014). While excessive ROS production leads to many metabolic complications in human health problems, including type II diabetes (Giacco and Brownlee, 2010), it has been increasingly recognized that physiological levels of ROS, mainly H_2_O_2_, play important roles in cellular functions that are linked with the regulation of several cellular activities, including cellular signaling, cell differentiation, and cell proliferation (Schieber and Chandel, 2014). Moreover, the physiological and local production of H_2_O_2_ is important in many signaling pathways, such as tyrosine phosphorylation, which controls kinase activity, including the insulin receptor (IR) kinase, protein kinase B (AKT), and phosphoinositide 3-kinase (PI3K). Moreover, the phosphatases, i.e., phosphatase and tensin homolog (PTEN), protein tyrosine phosphatase 1B (PTP1B), and protein phosphatase 2A (PP2A), which act via dephosphorylating the molecules in the insulin signaling, are also inhibited by the physiologically produced H_2_O_2_ (Lennicke and Cocheme, 2021a; Lennicke and Cocheme, 2021b). Therefore, the concentrations and spatiotemporal productions of physiological levels of ROS significantly impact the activation of the insulin signaling cascade.

Previous studies reveal that controlled ROS production is essential to conserve several important functions, such as cell host-defense mechanism, proliferation, cell signaling, etc (Nita and Grzybowski, 2016; Droge, 2002). The mitochondrial electron transport chain (ETC) generates superoxide free radicals by the single-electron drip at complexes I and III of the oxidative phosphorylation pathways (Zorov et al., 2014; Murphy, 2009; Zhao et al., 2019; Quinlan et al., 2014). Under physiological conditions, a cellular equilibrium exists between ROS production and its clearance since eukaryotic cells possess numerous anti-oxidative cell protection machinery, including enzymes and antioxidants (Pizzino et al., 2017; Nita and Grzybowski, 2016; Juan et al., 2021). Five types of intracellular antioxidant enzymes called: (i) Cu/Zn-superoxide dismutase (Cu/Zn-SOD, SOD1) present in the cytosol, (ii) manganese superoxide dismutase (Mn-SOD, SOD2) present in the mitochondrial matrix, (iii) catalase, (iv) glutathione peroxidase (GPx), and (v) glutathione reductase. The SODs catalyze superoxide to oxygen and hydrogen peroxide (H_2_O_2_), while catalase and GPx convert H_2_O_2_ into H_2_O and O_2_ (Nita and Grzybowski, 2016). A basal level superoxide is stored in healthy individuals and is believed to be accountable for aging (McCord, 2000; Finkel and Holbrook, 2000; Schriner et al., 2005). Stress-induced and mitochondrial dysfunction-related ROS can pave the way to developing disease states, such as cancer (Zhang et al., 2022; Bekhet and Eid, 2021), rheumatoid arthritis (Wang et al., 2020), neurological disorders (Qu et al., 2019; Takahashi et al., 2019), pulmonary diseases (Siques et al., 2021), and diabetes mellitus (Adeshara et al., 2022; Singh et al., 2022; Chu et al., 2022).

However, ROS overproduction overwhelms intrinsic antioxidant capacity and may progress to damage the biomolecules of normal cells (Nita and Grzybowski, 2016; Droge, 2002). Increased ROS have long been considered dangerous for health and play key roles as signaling molecules by regulating several cellular states such as growth, differentiation, and apoptosis (He et al., 2017). Previous studies have reported that ROS can play distinctive, sometimes opposing roles conditional on their subcellular origin and abundance (Sun et al., 2020). Among ROS, H_2_O_2_ is the dominant cellular oxidant, and it is a significant contributor to redox regulation (Zorov et al., 2014).

## 2 Redox signaling and diabetes mellitus

**FIGURE 2:**
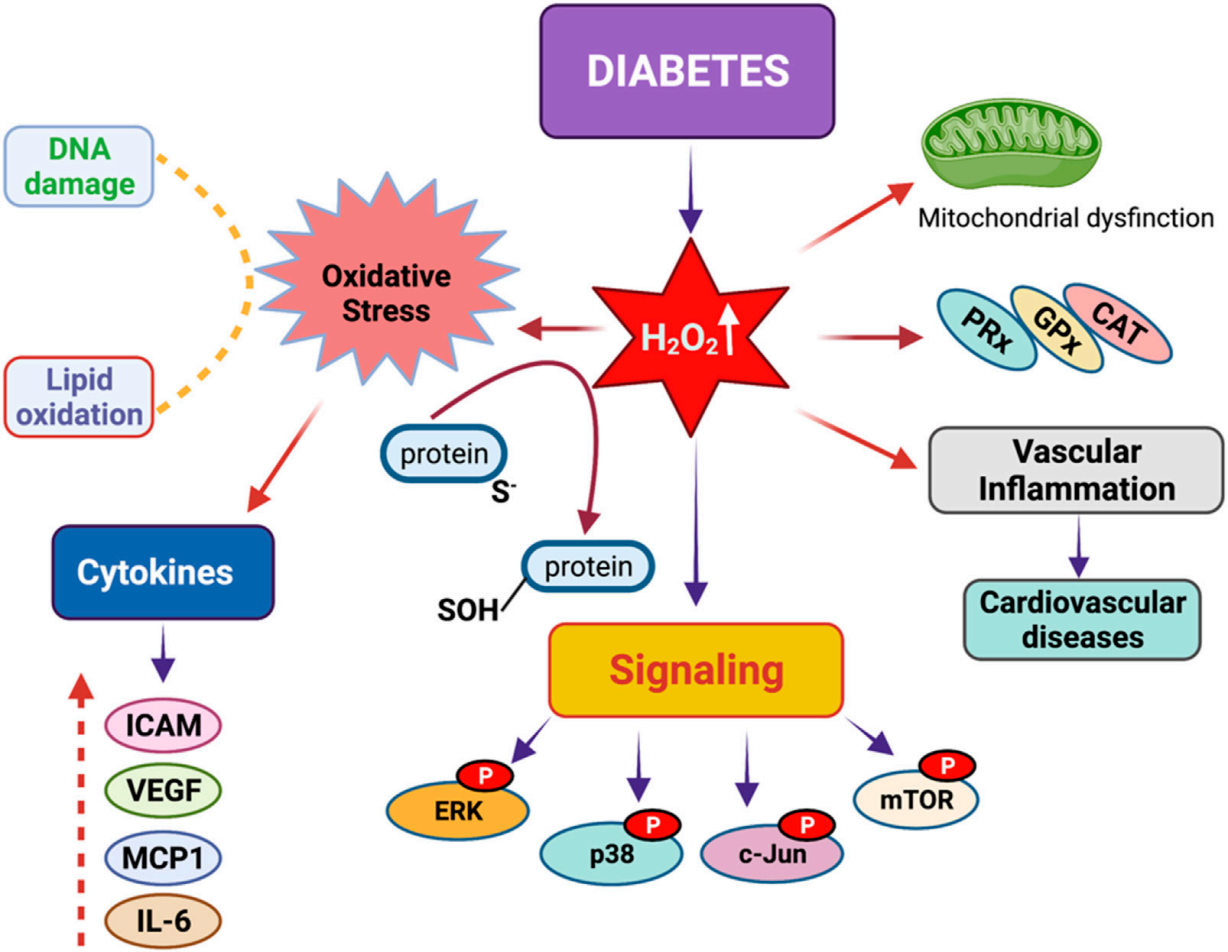
Redox signaling in diabetes mellitus. Diabetes mellitus leads to a significant increase in hydrogen peroxide concentrations in the body. Excess hydrogen peroxide can result in heightened oxidative stress, vascular inflammation, mitochondrial dysfunction, DNA damage, and issues with lipid peroxidation. These problems arise due to the phosphorylation of proteins such as ERK, p38, c-Jun, and mTORC1, which disrupt key signaling pathways by altering the levels of various cytokines, including ICAM, VEGF, MCP1, and IL-6.

Hyperglycemia causes tissue damage through the induction of multiple pathways, including active polyol pathway and boosted intracellular glycation end-products (AGEs) that ultimately induce the expression of AGE receptors and activation of protein kinase-C isoforms (Giacco and Brownlee, 2010; Wu et al., 2018). Many studies have demonstrated that alterations in cellular redox and excess production of oxidative stress play a key role in the development of diabetes mellitus (Lennicke and Cocheme, 2021b; Korac et al., 2021; Rodriguez Camargo et al., 2017; Delmastro and Piganelli, 2011; Manda et al., 2015; Jha et al., 2016; van Dijk et al., 2020; Chang and Chuang, 2010; Chaudhuri et al., 2018). The elevated level of energy substrates and the inflammatory environment with excess calorie uptake result in the overproduction of mitochondrial ROS that suppresses insulin signaling and leads to the development of insulin resistance (Chang and Chuang, 2010). Under diabetic conditions, persistent hyperglycemia causes an oversupply of NADH through mitochondrial complex I, which produces more superoxide, precursors of all ROS (Giacco and Brownlee, 2010; Murphy, 2009; Yan, 2014a; Wu et al., 2016; Laustsen et al., 2017; Yan, 2014b; Bhatt et al., 2015). Figure 2 demonstrates the roles of diabetes-related ROS in the alterations of cellular signaling.

Redox signaling is a major factor in wound healing processes in diabetes (Kunkemoeller and Kyriakides, 2017; Jones et al., 2019). Diabetic ulcers are caused by damage to the peripheral vascular system, inflammation, elevated protease levels, and an aberration in matrix metalloproteinase (MMP) levels in tissues (Blakytny and Jude, 2006). Amplified mitogenic action and suppressed protease activity are principal therapeutic approaches for the management of wound healing in diabetes (Baltzis et al., 2014). Contrasting to proteases, angiogenic factors, such as hypoxia-inducible factor 1α (HIF1α), have a key role in repairing wound lesions in diabetic tissues (Hunt et al., 2007). Several reports demonstrated that hypoxia inhibits MMP2 and MMP9 expression (Ye et al., 2014; Rahat et al., 2006; Liu et al., 2018). HIF1α can accelerate extracellular matrix (ECM) deposition in injured tissues (Gilkes et al., 2013) and tissue fibrosis (An et al., 2004). Along with hypoxia, ROS can also efficiently trigger an elevated level of HIF-1α expression (Peng et al., 2006). In addition, the malondialdehyde (MDA) level, which is used to predict lipid peroxidation, is higher in the diabetic group than in the control group. In contrast, the total thiol was lower or no change in diabetes compared to nondiabetic controls (Modaghegh et al., 2021; Esteghamati et al., 2013). Besides, the reduction of nitric oxide (NO) generation in diabetes also hampers wound healing with lowered angiogenesis and extracellular matrix (ECM) deposition (Soneja et al., 2005).

The complications associated with diabetes are pivotal in determining the quality and mortality of individual life with diabetes. Under diabetic conditions, increased oxidation of fatty acids due to decreased glycolysis leads to the overproduction of ROS, which causes damage and dysfunction of many tissues and organs with elaborated vascular systems, such as the retina and kidney. Overproduction of ROS by mitochondria leads to other complications in humans (Guo et al., 2013). However, in diabetes, ROS overproduction seems to result from increased oxidation of fatty acids due to insulin resistance (Chawla et al., 2016). The ECM function and structure are damaged in diabetes due to fibroblast dysfunction and protein deposition alterations, which ultimately lead to abnormal ECM structure and composition (Law et al., 2012). Diabetic nephropathy (DN) is a primary complication that leads to end-stage renal disease across the globe (Fu et al., 2019). Hyperglycemia-induced injuries in the epithelial cells have been recognized to several key mechanisms, including increased ECM deposition, generation of advanced glycation end products, and activated canonical Wnt signaling (Duni et al., 2019; Hu et al., 2015; Kanwar et al., 2011). Recent studies reported that mitochondrial ROS is amplified in db/db mice kidneys; this study also demonstrated that a redox-sensitive green fluorescent protein biosensor (roGFP) in real-time *in vivo* and found that the biosensor was obviously expressed in the mitochondrial matrix of db/db mt-roGFP mouse (Galvan et al., 2017; Han et al., 2018). Furthermore, several studies have demonstrated that excessive mitochondrial ROS production triggers apoptosis and tubular cell injury in diabetic states (Han et al., 2018; Awad et al., 2015; Sun et al., 2010). Hence, mitochondrial ROS overproduction plays a crucial role in tubular damage in diabetic nephropathy.

Metformin is the most widely used oral anti-diabetic agent (Nassif et al., 2022; Viollet et al., 2012). Several *in vivo* studies have shown that metformin reduces hydroxyl free radicals and ROS production in bovine aortic endothelial cells (Nassif et al., 2022; Sivitz and Yorek, 2010). Metformin considerably decreases levels of urinary F_2_-isoprostanes and increases vitamins A and E in plasma in type 2 DM patients. *In vitro* experiments have suggested that metformin prevented the development of advanced glycation end-moieties by activating AMPK levels (Ruggiero-Lopez et al., 1999; Kanasaki et al., 2017). Metformin treatment has been shown to obstruct the development of NAD(P)H oxidase and, thus, lower hydrogen peroxide production i*n* cultured endothelial cells under hyperglycemic conditions (Gallo et al., 2005). Sulfonylureas release insulin granules through binding to the ATP-dependent K^+^ (K_ATP_) channel complex on the cellular transmembrane of β-cells, increasing intracellular calcium levels and releasing proinsulin. Gliclazide, a class of sulphonylurea, and a study of 44 Type two diabetic mellitus subjects using Gliclazide for the duration of 10 months resulted in a noticeable reduction of 8-isoprostanes, a key marker of lipid oxidation, and an elevated level of the total antioxidant capability (McGavin et al., 2002). Other sulfonylureas, including Glipizide, Tolazamide, and Glibenclamide, have marginal outcomes on antioxidant action (Kalra et al., 2022). Thiazolidinediones (TZD) counteract insulin resistance through its binding to PPARγ, which activates the transcription of antioxidant-associated genes, including superoxide Dismutase1 (SOD) and catalase (CAT) (Khoo et al., 2013). Statins work competitively by hindering HMG-CoA reductase and lowering liver cholesterol synthesis (Wassmann et al., 2002). The effects of anti-diabetic drugs on cellular redox were summarized in Table 1.

**TABLE 1:** Describes the effect of antidiabetic drugs on the action of ROS.

Drugs	Action on ROS	Cell type	Disease
Metformin	↓ Urinary F2 isoprostanes ↑ Vitamins A and E in plasma ↓ NADPH oxidase ↓ Hydrogen peroxide	Endothelial	Diabetes, Hyperglycemia
Sulfonylureas	↑ Intracellular calcium levels ↑ Release poroinsulin	Pancreatic beta cells	Diabetes
Thiazolidinediones	↑ Transcription SOD, CAT	Adipocytes	Diabetes
Atorvastatin	↓ mRNA expression nox1 ↓ translocation rac1 GTPase	Smooth muscle, Endothelial, T Cells	Heart disease, Stroke, Diabetes

## 3 Vascular dysfunction in diabetes

Altered redox signaling in the diabetic endothelium is a critical regulator of vascular disease pathogenesis. Elevated levels of ROS in the endothelium have been associated with vascular disease pathogenesis (Incalza et al., 2018; Chen et al., 2018). The detrimental effects of hyperglycemia on vascular biology have been studied intensively at preclinical and clinical levels. However, vascular redox state regulation mechanisms and their clinical relevance have not been well studied in diabetic conditions (Yang et al., 2024; Xu and Zou, 2009). Vascular endothelial cells play critical roles in the regulation of cardiometabolic health (Li et al., 2023). Endothelial cells secrete diverse regulatory molecules that control platelet aggregation, fibrinolysis, and vascular tone (Avogaro et al., 2011). Endothelial cell dysfunction is characterized as a condition in which the endothelial cells lose their features, including the ability to promote vasodilation, anti-aggregation, and fibrinolysis (Zhang et al., 2024). Endothelial cell mediators, i.e., endothelin-1 and thromboxane A2, promote vasoconstriction, and mediators, for example, NO, prostacyclin, and endothelium cell-derived hyperpolarizing factor, promote vasodilation (Avogaro et al., 2011). The endothelium has a restricted intrinsic ability for self-repair, and endothelial cell repair is linked with and accomplished through the contribution of endothelial progenitor cells in the physiological and diabetic milieu (Avogaro et al., 2011).

In diabetes, coronary circulation shows an accumulative level of acetylcholine (Ach), which causes contraction (Nitenberg et al., 1993). The contraction stimulated by Ach is facilitated over the M3 subtype of muscarinic receptors and loss of endothelial cell integrity, suggesting that endothelial cells in hyperglycemia undergo an apoptotic process, causing intimal denudation. Interestingly, acetylcholine has been shown to cause both vasoconstriction and vasodilation in coronary vessels. The more common effect is the dilation of healthy blood cells when muscarinic M3 receptors are stimulated (Gericke et al., 2011; Tangsucharit et al., 2016; Liu et al., 2024). Due to the release of nitric oxide, vascular smooth muscle relaxes, resulting in vasodilation (Chataigneau et al., 1999). This effect will commonly be seen in large arteries and coronary vessels while under normal conditions; however, damaged endothelial tissue due to inflammation and atherosclerosis can result in differing effects (el-Tamimi et al., 1993). Due to impaired nitric oxide production in damaged vessels, acetylcholine can paradoxically result in vasoconstriction of blood vessels when the M3 receptors are stimulated (Gericke et al., 2014). Intracoronary acetylcholine is generally known to cause vasodilation, resulting in increased blood flow, although in large coronary arteries, it has been shown to cause vasoconstriction. Thus, individual endothelial health as well as the classification of the specific coronary vessel, will greatly impact whether acetylcholine results in vasodilation or vasoconstriction. In addition, the integrin-β1 signaling is an initiator of apoptosis events, which later phosphorylates mitogen-activated protein kinase and c-Jun N-terminal protein kinase proteins. Suppression of endothelial cadherin activates the caspase proteins, resulting in apoptosis. A study has shown higher levels of circulating endothelial cells in type 2 diabetic patients (McClung et al., 2005). Besides circulating endothelial cells, microparticles (endothelial microparticles) may also play a role in procoagulant activity. A higher level of endothelial cell-derived endothelial microparticles is a prognostic feature to detect the presence of coronary artery lesions. This has substantial independent consequences than the diabetes states, lipid levels, hypertension, etc. (Nomura, 2009). The magnitude of the apoptosis is called arterial denudation, which elicits critical pro-atherosclerotic mechanisms, including smooth muscle cell proliferation, migration, and ECM secretion in diabetic conditions.

**FIGURE 3:**
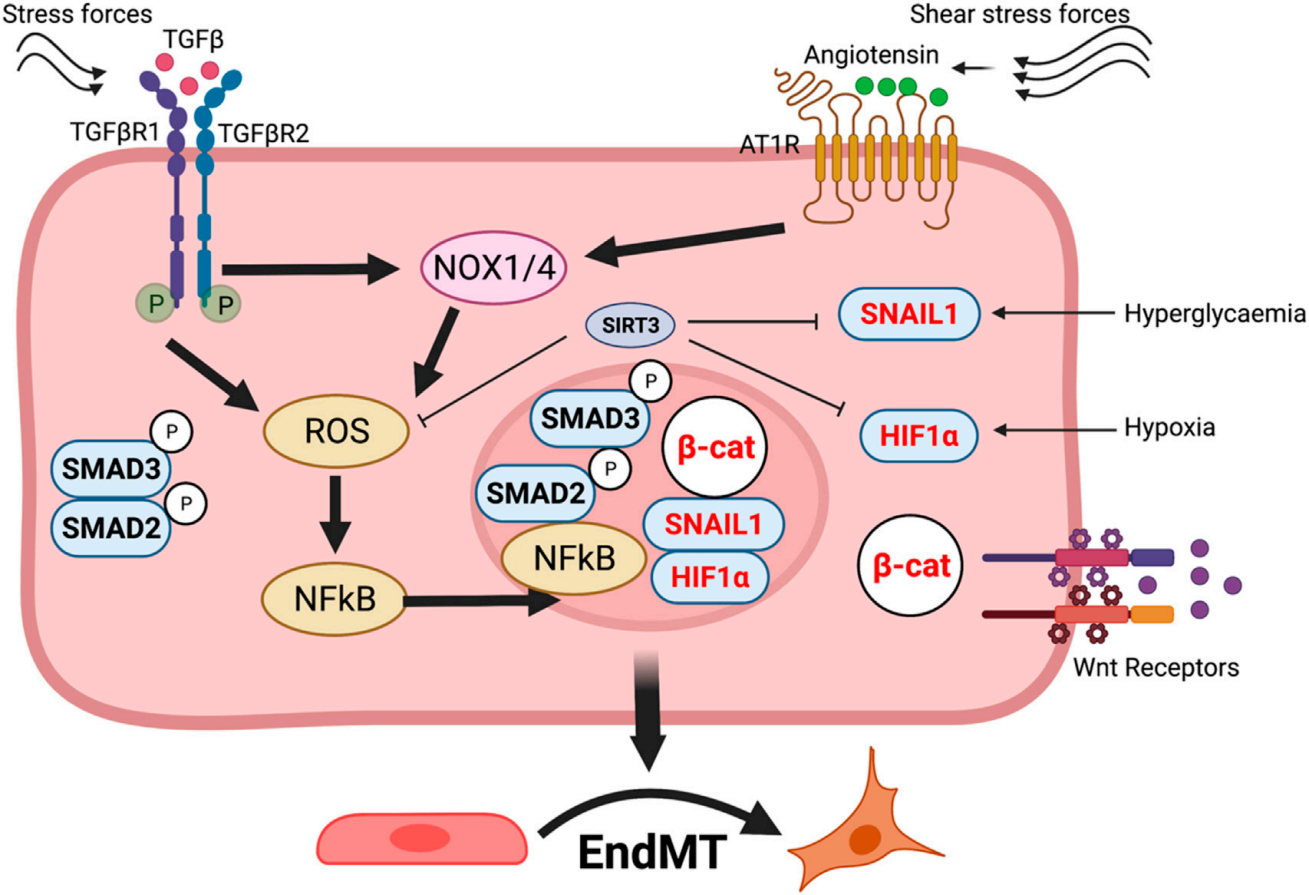
Redox signaling in diabetic endothelium. Under diabetic condition, the increased dimerization of TGFβR1 and TGFβR2 leads to the phosphorylation of Smad2 and Smad3, producing ROS activating NF-kB. Phosphorylated Smad2 and Smad3 along with NF-kB, then enter the nucleus. Additionally, in diabetes, elevated Wnt signaling and hypoxia contribute to increased levels of β-catenin, accumulation of HIF1α, and expression of Snail1 in the nucleus. The combined effects of NF-kB, Smad2, Smad3, HIF1α, Snail1, and β-catenin in the nucleus drive the transcription of mesenchymal and inflammatory genes. This results in mesenchymal cellular reprogramming in endothelial cells or EndMT under diabetic conditions.

In diabetes, endothelial cells can transdifferentiate into other intermediate cell types with endothelial cell markers and smooth muscle (SM)-like cells (Srivastava et al., 2013; Nagai et al., 2014) and mesenchymal cell phenotypes (Srivastava et al., 2013; Nagai et al., 2014; Srivastava et al., 2021a; Li et al., 2017; Shi et al., 2015). TGF-β signaling, Wnt signaling, DPP-4 signaling, and hedge-hog signaling play a key function in the mesenchymal activations in the endothelium (Srivastava et al., 2019; Kanasaki et al., 2014; Srivastava et al., 2020a). Medici et al. demonstrated cultured endothelial cells expressing integral effective activin-like kinase-2 display an endothelial-to-mesenchymal transition (EndMT) and acquire a stem cell-like phenotype (Medici et al., 2010). Either TGF-β2 or BMP4-based treatment induces trans-differentiation of endothelial cells to the intermediated type of cells, later the stem-like cells (Medici et al., 2010). Notably, EndMT is known to contribute to cardiac fibrosis, which leads to progressive ventricular wall thickening, adipose tissue fibrosis, and renal fibrosis (Goumans et al., 2008; Zeisberg et al., 2007a; Zeisberg et al., 2007b; Srivastava et al., 2014). Thus, vascular endothelial cells have substantial plasticity, which may highly correlate to the structural and functional role of endothelial cells. However, the mechanisms by which redox governs the endothelial cell-lineage switch remain elusive. Moreover, hyperglycemia-associated ROS and H_2_O_2_ are linked to activation of EndMT through cytokine reprogramming and phosphorylation of signaling such as extracellular signal-related kinase (ERK), Jun, mTOR, and p38, and nuclear accumulation of phosphorylated smad2/3, NF-kB, HIF1α, β-catenin, and Snail1. The orchestrated and cumulative effects of these pathways result in cellular reprogramming favoring mesenchymal transitions (Figure 3).

When there is an excess amount of ROS present in the body, the activity of ion channels can be negatively impacted, resulting in misbalances within smooth and endothelial cells (Ramirez et al., 2016). Through oxidative modifications, channel proteins can experience conformation alterations that affect their gating properties (Kourie, 1998). In smooth muscle cells, calcium channel activity can be enhanced, resulting in increased intracellular calcium, while potassium channels are inhibited, leading to sustained vasoconstriction (Gorlach et al., 2015; Tykocki et al., 2017). Moreover, ROS-mediated signaling can alter kinase pathways that phosphorylate ion channels, inducing further changes to function (Zhang et al., 2016). In diabetic conditions, damaged potassium and calcium channels promote endothelial dysfunction, while sodium imbalances caused by ROS-damaged channels can worsen endothelial permeability (Tykocki et al., 2017). In the end, ROS-damaged ion channels will significantly impact endothelial cell integrity as well as vascular ability.

Peroxynitrite is a highly reactive species that can form as a result of excessive ROS production due to diabetes (Zou et al., 2004). When not properly dealt with, excessive peroxynitrite formation can result in increased oxidative stress and vascular inflammation (Esposito and Cuzzocrea, 2009). Due to its ability to modify tyrosine residues, peroxynitrite has been shown to disrupt enzymatic function as well as inter-cellular signaling (Liaudet et al., 2009; Bartesaghi and Radi, 2018). By modifying signaling cascades, important pathways such as insulin signaling and ion channels can no longer function efficiently, further impairing cellular and vascular function. Within endothelial cells, peroxynitrite compromises structural integrity, hurting structures such as the blood-brain barrier, and can result in apoptosis (Tan et al., 2004; Dickhout et al., 2005).

## 4 AMPK and antioxidant regulation in diabetic endothelial cells

### 4.1 AMPK regulation in the healthy endothelial cells

**FIGURE 4:**
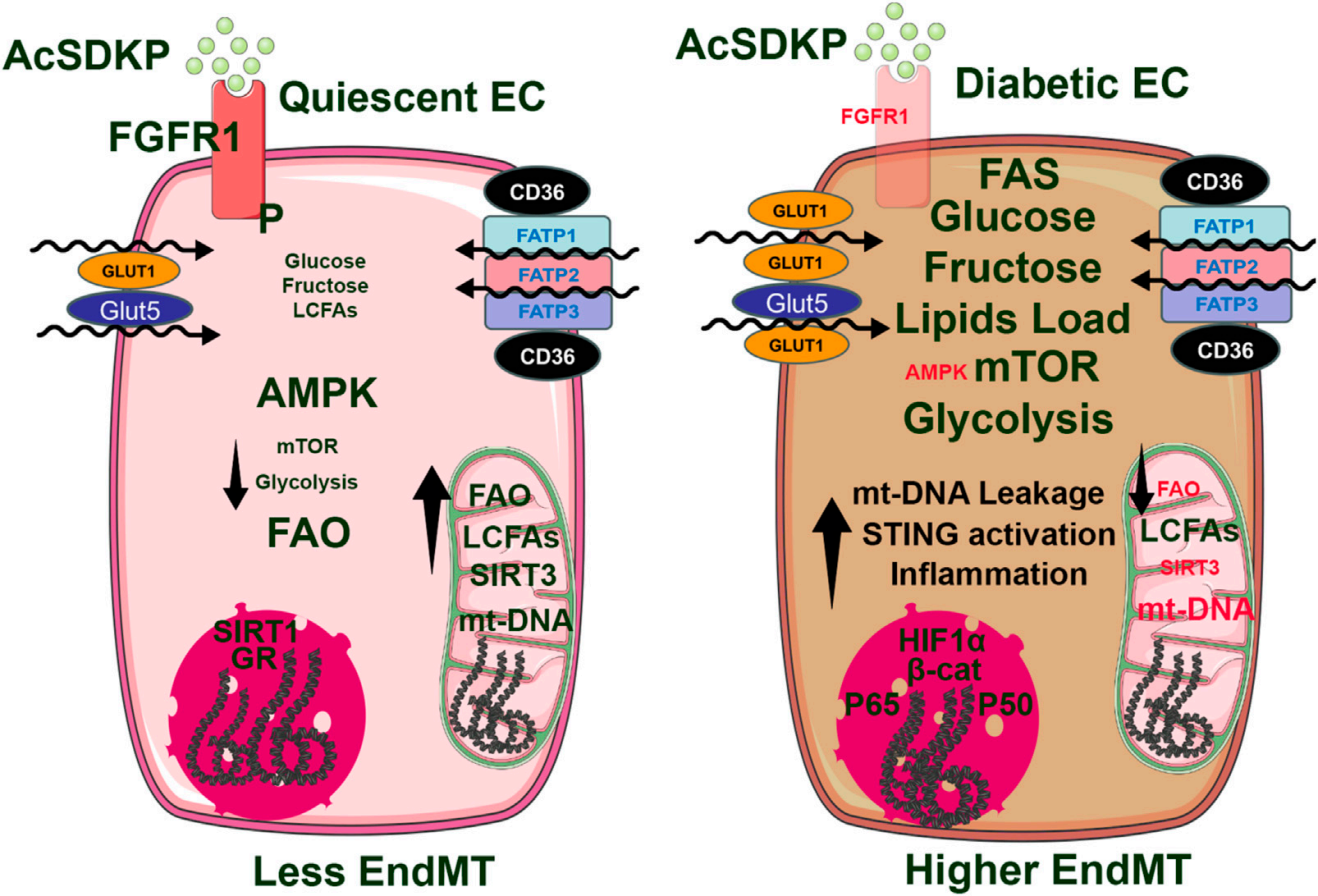
In normal, quiescent endothelial cells, high levels of AMPK expression are associated with low mTORC1 activity, reduced glycolysis, and increased fatty acid oxidation (FAO). This suggests that AMPK is a key kinase that promotes FAO and helps maintain mitochondrial integrity, which controls inflammation at lower levels and a reduced endothelial-to-mesenchymal transition (EndMT). Conversely, in diabetic endothelial cells, AMPK activity is diminished while mTORC1 activity is enhanced. This shift leads to increased glycolysis and decreased FAO, resulting in mitochondrial damage and leakage of mitochondrial DNA into the cytosol. These combined effects activate the cGAS-STING pathway, allowing the p65 and p50 subunits of NF-κB to enter the nucleus. This process is primarily responsible for the transcription of inflammatory genes, contributing to inflammation in endothelial cells and promoting the pro-EndMT signal.

In healthy endothelial cells, AMP-activated protein kinase (AMPK) is activated by cellular stressors, such as low energy levels, and plays a key role in cellular metabolism by promoting fatty acid oxidation (FAO), glycolysis, and autophagy (Lin et al., 2015), (Figure 4). AMPK activation enhances the expression of antioxidant enzymes, such as superoxide dismutase, NADH oxidase, and xanthine dismutase (Lin et al., 2015). These enzymes help convert reactive oxygen species (ROS) to hydrogen peroxide, which is subsequently reduced to water, thereby protecting cells from oxidative stress and preventing multiple cellular components from oxidative damage (Lin et al., 2015). In addition to directly upregulating antioxidant genes, AMPK activation controls NADPH levels by promoting FAO and inhibiting fatty acid synthesis (FAS) (Desjardins et al., 2022). NADPH serves as a critical reducing agent essential for regenerating antioxidants such as glutathione, which helps neutralize ROS and maintain cellular redox. AMPK activation in normal endothelial cells triggers the activation of several sirtuin proteins (SIRTs), particularly SIRT1 and SIRT3 (He et al., 2021; Marin et al., 2017), NAD-dependent type III histone deacetylases that also deacetylate many acetylated metabolic enzymes and other proteins. AMPK is known to increase mitochondrial β-oxidation to increase the NAD^+^/NADH ratio (Xu et al., 2009). Furthermore, AMPK-dependent phosphorylation of NAMPT enhances its activity to generate NAD^+^ from phosphoribosyl pyrophosphate (PRPP) (Liao et al., 2022). SIRT1 is highly expressed in endothelial cells, and active SIRT1 mitigates TGF-β-induced EndoMT by deacetylating transcription factors such as Smad4, which would otherwise promote endothelial-to-mesenchymal transitions (EndMT) (Liu et al., 2019). EndMT is marked by the loss of endothelial structure, preventing cells from promoting vasodilation or anti-aggregation (Xiong et al., 2018). SIRT3, another sirtuin primarily expressed in the mitochondria, is also activated by AMPK. SIRT3 deacetylates many mitochondrial proteins, including enzymes involved in the TCA cycle, ETC members, antioxidant defenses, and mtDNA repair, and maintains physiological mitochondrial function and metabolic pathways (Pillai et al., 2016; He et al., 2019; Hirschey et al., 2010; Koyama et al., 2011; Morigi et al., 2015; Bindu et al., 2017). SIRT3 deficiency in endothelial cells stimulates the TGFβ/Smad3-dependent mesenchymal transformations in renal tubular epithelial cells (He et al., 2019). In addition, SIRT3 also plays an important role in maintaining lipid and glucose metabolism and suppressing EndMT-mediated activation of the fibrogenic pathways in diabetic kidneys (Srivastava et al., 2021b). Ablation of SIRT3 in endothelial cells leads to metabolic shifts in myofibroblasts in diabetic kidneys (He et al., 2019; Srivastava et al., 2021b). Through this mechanism, AMPK ensures that endothelial cells maintain their normal phenotype, maintaining vascular stability and function (Han et al., 2021). AMPK also plays a key role in the regulation of nitric oxide synthase (NOS) activity (Rodriguez et al., 2021). In healthy kidney cells, AMPK promotes the phosphorylation of endothelial nitric oxide synthase (eNOS), which produces nitric oxide (NO), a key molecule in regulating blood pressure and supporting vascular tone (Ministrini et al., 2021). Nitric oxide produced by eNOS plays an important role in vasodilation and anti-inflammatory responses. In contrast, inducible nitric oxide synthase (iNOS) is expressed mainly during inflammatory responses and produces larger amounts of NO, which can contribute to oxidative stress if not properly regulated. Under normal metabolic conditions, endothelial cells express CD31, a platelet-endothelial cell adhesion molecule, and vascular endothelial (VE) cadherins, which support endothelial integrity and function. Low levels of mesenchymal markers such as vimentin and fibroblast-specific protein (FSP1) indicate minimal EndMT and reflect stable endothelial phenotype. Glutamine, the most abundant amino acid in the bloodstream, undergoes glutaminolysis to form alpha-ketoglutarate, an intermediate of the TCA cycle, sustaining ATP levels as cellular energy and thus mitigating AMPK activation (Bodineau et al., 2022; Bodineau et al., 2021; Li et al., 2024). AMPK activation inhibits aberrant angiogenesis by inhibiting pathways such as TGF-β, which is a major regulator of blood vessel formation (Zhao et al., 2021). TGF-β activates the BMP/Smad1/5/8 pathway, which upregulates Id proteins (Id1-3) (inhibitors of differentiation) that inhibit mesenchymal transition in the endothelial cells and help maintain endothelial cell differentiation, sprouting, proliferation, and angiogenesis (Ma et al., 2021). The balance of tip and stalk endothelial cell phenotypes, regulated by Smad1/5/8, allows for orderly angiogenesis. Disruption in Smad1/5-Notch signaling can lead to imbalanced vessel growth, excessive sprouting, and migration issues, which are avoided under normal conditions where AMPK is capable of active and functioning as a stabilizer (Deng et al., 2024).

### 4.2 AMPK regulation in diabetic endothelial cells

Under diabetic conditions, AMPK activation is significantly reduced, leading to impaired fatty acid oxidation (FAO) and glycolysis (Ren and Shen, 2019), (Figure 4). This metabolic shift results in decreased expression of antioxidants that AMPK would normally upregulate, making cells more susceptible to oxidative stress (Marino et al., 2021). Excessive ROS generated in mitochondria damages cellular components and interferes with insulin signaling, which contributes to insulin resistance, a hallmark of diabetes. Due to reduced FAO, lower levels of NADPH limit cells’ ability to regenerate antioxidants, such as glutathione, compounding oxidative stress and cellular vulnerability. With reduced AMPK activity, there is decreased activation of SIRT1, weakening its inhibition of TGF-β-induced EndMT in diabetic kidney endothelial cells (Liu et al., 2019). This loss of inhibition allows TGF-β signaling to promote the transformation of endothelial cells into a mesenchymal phenotype, contributing to endothelial dysfunction and tissue fibrosis (Tombor et al., 2021). Studies, such as those by Srivastava et al., demonstrate that the absence of SIRT3 in endothelial cells further drives their EndMT with increased FSP-1 and α-SMA expression while reducing CD31 expression. This shift indicates a progression from a healthy endothelial phenotype to a pro-fibrotic mesenchymal phenotype in the kidney of diabetic mice (Srivastava et al., 2021b). In diabetic kidney endothelial cells, reduced AMPK activity leads to lower eNOS phosphorylation, which impairs nitric oxide (NO) production (Li et al., 2023) and causes increased vascular stiffness, a common feature in diabetes (Li et al., 2023). Additionally, lower AMPK activity reduces the conversion of eNOS to iNOS, limiting NO production during inflammatory responses (Li et al., 2023; Tran et al., 2022). This deficiency in NO production contributes to an increased susceptibility to EndMT, as NO typically helps maintain the endothelial phenotype and prevents excessive mesenchymal transformation (Tran et al., 2022; Smeda et al., 2018; Islam et al., 2021). Under diabetic conditions, aberrant glutamine metabolism further impairs AMPK activation (Bodineau et al., 2021). Impaired nitrogen metabolism due to glutamine imbalance increases proteinuria, promoting kidney inflammation and fibrosis (Cai et al., 2024). Proteinuria also inhibits extracellular matrix (ECM) breakdown, leading to ECM accumulation, glomerular injury, and renal fibrosis, worsening diabetic kidney disease (Cai et al., 2024). The deprivation of glutamine metabolism *in vitro* and *in vivo* inhibits fibroblast activation, thereby suppressing renal fibrosis (Cai et al., 2024). In addition, glutamine metabolism is important for maintaining mitochondrial function and morphology. These effects partially depend on the metabolic intermediate α-ketoglutaric acid (Cai et al., 2024). Moreover, glutamine deprivation leads to upregulated mitochondrial fission in fibroblasts by activating the dynamin-related protein 1 pathway, suggesting that the defective glutamine metabolism initiates the regulation of mitochondrial function, thereby facilitating the progression of renal fibrosis. Targeting glutamine metabolism emerges as a novel and promising avenue for therapeutic intervention and prevention of renal fibrosis (Cai et al., 2024). In another study, it has been shown that in diabetic endothelial cells, low AMPK levels fail to inhibit TGF-β-induced angiogenesis, allowing unchecked Smad1/5/8 signaling that disrupts the balance of endothelial tip and stalk cells. This imbalance leads to aberrant vessel sprouting, abnormal migration patterns, and destabilized vascular growth (Ruderman et al., 2013; Mao et al., 2022). Reduced AMPK activation also decreases autophagy and VEGF expression, further impairing angiogenesis and increasing cellular invasion and migration, contributing to the formation of leaky vessels and defective vascular structures in diabetic tissues (Park et al., 2023; Madonna et al., 2023; Xie et al., 2011; Yao et al., 2016).

## 5 mTORC1 and oxidative signaling in diabetic endothelial cells

### 5.1 mTORC1 signaling and oxidative signaling in endothelial cells

The mechanistic/mammalian target of rapamycin (mTOR) is an evolutionarily conserved serine/threonine kinase found in all cells and tissues. mTOR plays a critical role in stimulating essential cellular anabolic processes, including protein, lipid, and nucleotide biogenesis, whereas it inhibits catabolic processes such as autophagy and lysosome biogenesis (Panwar et al., 2023). With these biochemical functions, mTOR acts as a central regulator of cell growth, proliferation, differentiation, and survival. Similar to the effect of oxidative stress-associated H_2_O_2_, physiological levels of mTOR activity are essential for supporting normal development and maintaining a healthy metabolism, whereas aberrant activation of mTOR leads to many human health problems, including cancer, metabolic disorders, and neurodegenerative diseases (Panwar et al., 2023).

mTOR forms two distinct functional complexes, known as mTORC1 and mTORC2. Both complexes have their unique function of phosphorylating their specific substrates. While mTORC2 is mainly activated by growth factors, mTORC1 receives inputs from both growth factors and nutrients for its activation. mTORC1 promotes anabolic processes, including the production of protein, lipids, and nucleotides. Along with this synthesis, mTORC1 inhibits catabolic processes, such as autophagy and β-oxidation of fatty acids (Kim and Guan, 2015). mTORC2, however, regulates cytoskeletal dynamics and metabolism (Kim and Guan, 2019). Especially, mTORC2 promotes glucose metabolism by activating Akt, a multifunctional kinase that stimulates glucose uptake into the cell (Panwar et al., 2023). Overall, mTOR kinase functions as a master regulator of cell growth, proliferation, survival, and metabolism in response to multiple environmental cues.

In endothelial cells and the vascular system, mTOR has a variety of functions as it stimulates angiogenesis and maintains the blood-brain barrier (Fingar et al., 2004; Tsang and Zheng, 2018; Wang et al., 2016). mTORC1 activity is key to producing vascular endothelial growth factor (VEGF) and nitric oxide (NO) (Wang et al., 2016; Wang et al., 2022). NO dictates vascular tone and plays an important role in maintaining blood vessel health by inhibiting leukocyte adhesion to the vascular wall and relaxing vascular smooth muscle (Wang et al., 2022; Glaviano et al., 2023).

While it has been reported that oxidative stress enhances cellular mTORC1 activity (Sarbassov et al., 2006), levels of cellular mTORC1 activity are also involved in the redox equilibrium and are required for its precise balance to prevent endothelium from oxidative stress (Li et al., 2010). The activation of mTORC1 increases mitochondria-derived ROS by enhancing mitochondria biogenesis and oxidative phosphorylation in the mitochondria. mTORC1 activation also leads to the reduction of mitophagy, causing the accumulation of damaged mitochondria, which often leak ROS into the cells (Kim and Guan, 2019; Li et al., 2010). Additionally, mTORC2-induced Akt activation can be attributed to the assembly of the NOX enzyme complex. The NOX complex is responsible for the production of ROS; thus, mTORC2 activation further increases rates of ROS production in the cell (Wang et al., 2022). Similarly, mTORC1, primarily via the PI3K/Akt pathway, also enhances the expression and membrane localization of GLUT1 in endothelial cells, promoting glucose uptake (Glaviano et al., 2023). With increased membrane GLUT1 expression, endothelial cells undergo more glycolysis and glucose-dependent reactions, such as anaerobic respiration (Lu et al., 2021).

The expression of antioxidant genes such as superoxide dismutase and glutathione peroxidase are reliant on the nuclear factor E2-related factor 2 (Nrf2) pathway, a system that has been shown to be tied to mTORC1 (Ngo and Duennwald, 2022). The Nrf2 pathway is a defense mechanism that protects the cells from oxidative stress (Gureev et al., 2020; Bendavit et al., 2016). It is a transcriptional program that responds to environmental stressors by activating enzymes that detoxify and are antioxidants (Nguyen et al., 2009). The Nrf2 pathway is involved in many cellular processes, including mitigating inflammation by suppressing pro-inflammatory cytokines and regulating other inflammatory mediators. For instance, Nrf2-induced antioxidants support the process of wound healing by reducing oxidative stresses. It also acts as anti-diabetes by facilitating insulin secretion, which prevents hyperglycemia. The Nrf2 pathway also mitigates tumor metastasis and prevents neurodegenerative diseases such as Alzheimer’s, Parkinson’s, and Huntington’s diseases (Nguyen et al., 2009; Faheem et al., 2020; Ahmed et al., 2017). The Nrf2 pathway is activated in several ways. Interestingly, oxidative stress, such as H_2_O_2,_ stabilizes Nrf2 by inhibiting Keap1 through its oxidation of several cysteine residues. It has also been reported that several kinases, including AMPK, CDK5, and PERK, phosphorylate Nrf2 and activate its transcriptional activity by protecting Nrf2 from degradation or inducing its nuclear translocation (Ahmed et al., 2017; Liu et al., 2021; Joo et al., 2016; Cullinan et al., 2003; Jimenez-Blasco et al., 2015). The Nrf2 pathway increases mTOR activity by enhancing its upstream signaling components, particularly through the PI3K/Akt pathway, allowing for coordinated cellular responses to environmental stressors (Gureev et al., 2020; Bendavit et al., 2016; Ji et al., 2023). When activated by oxidative stress, Nrf2 upregulates the expression of genes involved in the PI3K/Akt pathway, which subsequently leads to the activation of mTORC1 (Gureev et al., 2020; Bendavit et al., 2016; Ji et al., 2023). Moreover, Nrf2 has been shown to directly interact with the promoter region of the mTOR gene and enhance its transcription, or it can increase the transcription of upstream mTORC1 activators, such as RagD, a small GTPase protein that is crucial for lysosomal mTORC1 localization (Gureev et al., 2020; Bendavit et al., 2016; Ji et al., 2023). Understanding the Nrf2-mTOR crosstalk opens avenues for developing therapeutic strategies by targeting either pathway related to cellular responses to stress and disease conditions (Gureev et al., 2020; Bendavit et al., 2016; Ji et al., 2023).

The presence of varying levels of ROS in the cell has also been shown to potentially impact mTORC1 and AMPK activity, where low-to-moderate levels enhance mTORC1 signaling. In contrast, higher levels of ROS present can activate AMPK (Hinchy et al., 2018). Complementing this redox regulation includes sirtuin proteins, another class of antioxidants (Zhao et al., 2020; Zhao et al., 2017). SIRT1 deficiency increases mTORC1 signaling, while SIRT1 activators, such as resveratrol, reduce it (Ghosh et al., 2010). SIRT1 interacts with TSC2, a key enzymatic component of the TSC complex, to inhibit mTORC1 signaling (Ghosh et al., 2010). An increase in the expression of SIRT1 results in the downregulation of mTORC1 activity, resulting in the upregulation of the immunoglobulin CD31 (PECAM-1), an endothelial marker crucial for cell-cell adhesion and vascular integrity (Ghosh et al., 2010). SIRT1 deficiency promotes mesenchymal gain, such as increases in vimentin as well as α smooth muscle actin (α-SMA) expression, leading to EndMT (Liu et al., 2019; Piera-Velazquez et al., 2011; Mattagajasingh et al., 2007).

### 5.2 mTORC1 in the regulation of EndMT, inflammation, and fibrosis in diabetes

EndMT is a central step in many fibrotic diseases and even the development of cancer (Piera-Velazquez et al., 2011). While the direct link between cellular mTORC1 activity and EndMT remains elusive, mTORC1 activation may underlie the mechanisms of EndMT initiation. Under obese or diet-induced diabetic conditions, mTORC1 activity is generally upregulated in many organs and tissues. The enhanced mTORC1-S6K1 pathway leads to the activation of SREBP1, an essential transcription factor that stimulates lipid biogenesis (Bakan and Laplante, 2012). Elevated circulating free fatty acids (FFA) are accumulated in the endothelial cells, generating lipotoxicity, oxidative stress, and inflammation and promoting EndMT. The EndMT transition has many possible causes but is often a result of the presence of ROS and inflammation of the cell (Thuan et al., 2018; Lipke et al., 2022). Due to high lipid loads in the endothelial cells, mitochondrial damage and mitochondrial DNA (mt-DNA) leakage occur, which is then sensed by cyclic GMP-AMP synthetase (cGAS), stimulator of interferon genes (STING), and their downstream signaling adaptors, resulting in the translocation of p65 and p50 units of NF-_K_B into the nucleus and the activation of the transcription of genes important for the cellular inflammation (Chung et al., 2019). Under inflammatory conditions, the elevated FFAs lead to increased oxidative stress and inflammation in the endothelial cells, promoting EndMT (Chung et al., 2019; Srivastava et al., 2020b). A recent study from our laboratory demonstrates that angiopoietin-like 4 (ANGPTL4) is the critical regulator of lipid load and c-GAS-STING-related inflammation in diabetic tubules and podocytes (Srivastava et al., 2024). Defective lipid and glucose metabolism is associated with disruption of mitochondrial structure and functions and could be linked with the disruption in the redox signaling in the diabetic tubules and podocytes (Srivastava et al., 2024).

In addition, one possible connection to mTOR and increases in TGF-β signaling is seen through enhanced glutamine uptake because of high mTORC1. mTORC1 promotes this glutamine uptake through glutaminase (GLS) (Csibi et al., 2021). The increased influx of glutamine metabolism generates an energy-rich environment in the cell, supplying the necessary source for cells to make a potential transition (as seen in EndMT). Similarly, ROS has been shown to contribute to EndMT, suggesting potential crosstalk between glutaminolysis, ROS, and mTORC1 signaling in the initiation of EndMT (Thuan et al., 2018). In line with these, autophagy, which is inhibited by mTORC1, has been proposed to mitigate mesenchymal transitions, further supporting the link between mTORC1 and the initiation of EndMT (Kim and Guan, 2015).

## 6 New pharmaceutical agents and future directions

The imbalance of cellular redox status is a significant issue in diabetes and its related complications. In tissues that are insensitive to insulin, high glucose leads to an excess supply of NADH due to standard glucose oxidation and polyol pathway. In contrast, over-activation of Poly (ADP-ribose) polymerase (PARP) reduces the cellular NAD+ pool, which may downregulate sirtuin levels and further worsen the redox imbalance. Research into drugs that target aldose reductase in the polyol pathway (Yasunari et al., 2000) or PARP (Sarras et al., 2014) remain a promising area for future exploration. Furthermore, enzymes responsible for NAD+ regeneration, such as Complex I, should be investigated to understand how additional NADH can be oxidized in conditions of elevated glucose. NAD+ precursors can serve as a valuable alternative for addressing diabetes mellitus and its associated complications. The main objective of these potential therapeutic strategies is to restore the balance of NADH/NAD+ redox equilibrium in diabetes and related conditions.

**FIGURE 5:**
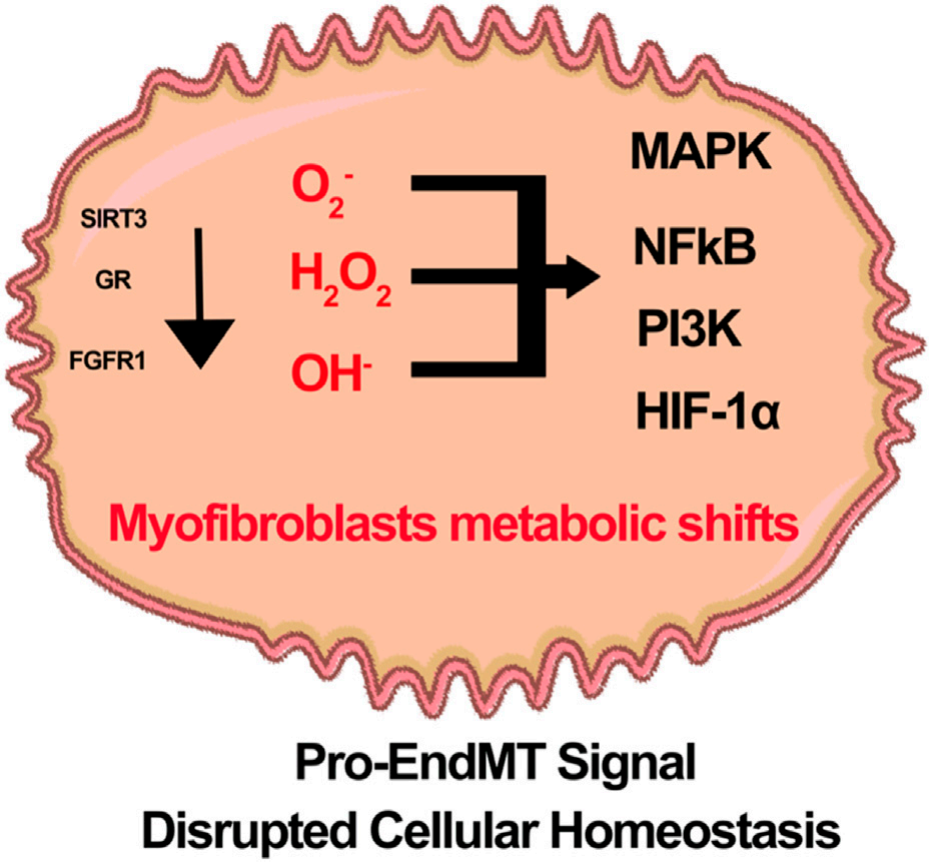
A hypothetical schematic diagram illustrates the role of Glucocorticoid receptor (GR), Sirtuin3 (SIRT3), and fibroblast growth factor receptor 1 (FGFR1) deficiency in the redox signaling and metabolic shift of myofibroblasts in diabetic endothelial cells. It is theorized that pro-EndMT signals disrupt cellular homeostasis and decrease the activity of SIRT3, GRs, and FGFR1. This disruption leads to an increased presence of reactive oxidative species. The defects in redox signaling in diabetic endothelial cells enhance a variety of inflammatory pathways, including MAPK, NF-kB, PI3K and HIF1α.

Recent studies indicate that nuclear glucocorticoid receptors (GR), mitochondrial sirtuin 3 (SIRT3), and fibroblast receptor growth factor 1 (FGFR1) play crucial roles in regulating endothelial cell senescence, integrity, cellular function, and metabolism (Srivastava et al., 2021b; Srivastava et al., 2021c; Li et al., 2020a; Srivastava and Goodwin, 2023). Endothelial GR deficiency accelerates aberrant Wnt signaling, increases IL-6 levels in plasma, and promotes defective FAO. Subsequently, endothelial cells exhibit a phenotype characteristic of EndMT. Intermediate cell types derived from EndMT can influence neighboring cells, ultimately contributing to renal fibrosis in diabetes (Srivastava et al., 2021c; Srivastava et al., 2021d). SIRT3 deficiency is linked to abnormal glycolysis and reduced FAO. These metabolic alterations are associated with the induction of mesenchymal characteristics in endothelial cells and neighboring epithelial cells, a process referred to as myofibroblast metabolic shifts (Srivastava et al., 2021b; Srivastava et al., 2018; Li et al., 2020b). Additionally, FGFR1 deficiency impairs the protective effects of N-acetyl-seryl-lysyl-proline (AcSDKP) in diabetic endothelial cells. This deficiency is associated with the downregulation of antifibrotic microRNAs, specifically miR-29 and miR-let-7b. As a result, there is an activation of profibrotic signaling pathways, including TGFβ signaling, DPP-4, and Integrin β1. These changes contribute to a mesenchymal transition in endothelial cells and renal tubules (Srivastava et al., 2020a; Li et al., 2020a; Srivastava et al., 2020c; Srivastava et al., 2016). The molecules associated with metabolic shifts and alterations in fuel preferences are linked to the activation of mesenchymal reprogramming. It is believed that hyperglycemia-related ROS and H_2_O_2_ play a significant role in the mesenchymal activation observed in states deficient in glucocorticoid receptor, SIRT3, or FGFR1. This suggests that GR, SIRT3, and FGFR1 are critical for lineage switching in endothelial cells (Figure 5). Further investigations are necessary to portray the cellular properties and translational potential of vascular redox conditions in relation to vascular disease in diabetes.
